# Overexpression of RsMYB1 Enhances Anthocyanin Accumulation and Heavy Metal Stress Tolerance in Transgenic Petunia

**DOI:** 10.3389/fpls.2018.01388

**Published:** 2018-09-20

**Authors:** Trinh Ngoc Ai, Aung Htay Naing, Byung-Wook Yun, Sun Hyung Lim, Chang Kil Kim

**Affiliations:** ^1^Department of Horticultural Science, Kyungpook National University, Daegu, South Korea; ^2^School of Agriculture and Aquaculture, Tra Vinh University, Trà Vinh, Vietnam; ^3^School of Applied Biosciences, Kyungpook National University, Daegu, South Korea; ^4^National Institute of Agricultural Science, RDA, Jeonju, South Korea

**Keywords:** abiotic stress, gene expression, genetic transformation, MYB transcription factor, phylogenetic analysis, plant growth

## Abstract

The RsMYB1 transcription factor (TF) controls the regulation of anthocyanin in radishes (*Raphanus sativus*), and its overexpression in tobacco and petunias strongly enhances anthocyanin production. However, there are no data on the involvement of RsMYB1 in the mechanisms underlying abiotic stress tolerance, despite strong sequence similarity with other MYBs that confer such tolerance. In this study, we used the anthocyanin-enriched transgenic petunia lines PM6 and PM2, which overexpress RsMYB1. The tolerance of these lines to heavy metal stress was investigated by examining several physiological and biochemical factors, and the transcript levels of genes related to metal detoxification and antioxidant activity were quantified. Under normal conditions (control conditions), transgenic petunia plants (T_2_-PM6 and T_2_-PM2) expressing RsMYB1, as well as wild-type (WT) plants, were able to thrive by producing well-developed broad leaves and regular roots. In contrast, a reduction in plant growth was observed when these plants were exposed to heavy metals (CuSO_4_, ZnSO_4_, MnSO_4_, or K_2_Cr_2_O_7_). However, T_2_-PM6 and T_2_-PM2 were found to be more stress tolerant than the WT plants, as indicated by superior results in all analyzed parameters. In addition, RsMYB1 overexpression enhanced the expression of genes related to metal detoxification [*glutathione S-transferase (GST)* and *phytochelatin synthase (PCS)*] and antioxidant activity [*superoxide dismutase (SOD)*, *catalase (CAT)*, and *peroxidase (POX)*]. These results suggest that enhanced expression levels of the above genes can improve metal detoxification activities and antioxidant activity, which are the main components of defense mechanism included in abiotic stress tolerance of petunia. Our findings demonstrate that RsMYB1 has potential as a dual-function gene that can have an impact on the improvement of anthocyanin production and heavy metal stress tolerance in horticultural crops.

## Introduction

Heavy metals occur naturally in the earth’s crust. However, excess levels of heavy metals produced by natural or anthropogenic activities are detrimental to living organisms. Over the past few decades, advances in industrialization and modern agricultural practices worldwide have led to contamination of cultivatable land with the heavy metals released from agro-chemicals and industrial activities (Yang et al., 2005). Generally, heavy metals, such as Zn, Cu, and Mn, play important roles in plant physiological and biochemical processes, such as chlorophyll biosynthesis, photosynthesis, and DNA synthesis (reviewed by Singh et al., 2015). For example, Zn contributes to the maintenance of membrane integrity, auxin metabolism, and reproduction because it interacts with enzymes and transcription factors (TFs) underlying these processes (Williams and Pittman, 2010; Prasad, 2012; Ricachenevsky et al., 2013). However, the toxic effects of heavy metals at high concentrations have also been well-documented (Fontes and Cox, 1998; Lewis et al., 2001; Warne et al., 2008; Ai et al., 2018). Zn at elevated concentrations can cease plant metabolic functions, causing growth retardation and senescence (Fontes and Cox, 1998; Warne et al., 2008). It has been reported that high Cu concentrations cause a similar range of symptoms (Lewis et al., 2001; Ai et al., 2018). In addition, elevated Mn concentrations are toxic to many plant species (Izaguirre-Mayoral and Sinclair, 2005; Rezai and Farboodnia, 2008), and high Cr levels negatively affect cell division and root and stem growth in many plants (Shanker et al., 2005; Zou et al., 2006; Fozia et al., 2008). Overall, elevated concentrations of these metals lower biomass accumulation and crop productivity by inhibiting several plant mechanisms. Theoretically, the presence of excess heavy metals limits CO_2_ fixation and reduces photosynthetic electron transport chains in chloroplasts and mitochondria. This leads to the overproduction of reactive oxygen species (ROS), which damage plant cells and inhibit plant growth, thereby reducing crop yields (Davidson and Schiestl, 2001; Mittler et al., 2004; Keunen et al., 2011). Therefore, it is important to understand how plants respond to heavy metal stress at physiological and molecular levels, and to develop plants that can resist stress-induced ROS overproduction and maintain crop productivity.

The roles of glutathione (GSH) and the phytochelatin synthase (*PCS*) gene in reducing heavy metal stress and ROS scavenging have been documented (Millar et al., 2003; Freeman et al., 2004; Foyer and Noctor, 2005; Hirata et al., 2005; Shao et al., 2008; Ai et al., 2018). The roles of antioxidants in scavenging ROS and reducing the oxidative stress caused by heavy metals have also been investigated (Hirschi et al., 2000; Tseng et al., 2007). As anthocyanin-enriched plants contain higher levels of antioxidants, which can effectively scavenge ROS, these plants are able to survive abiotic and biotic stress conditions (Winkel-Shirley, 2002; Dixon et al., 2005; Agati et al., 2011; Fini et al., 2011; Dehghan et al., 2014; Nakabayashi et al., 2014). Transgenic potato plants overexpressing *IbMYB1* (Cheng et al., 2013) and transgenic tobacco plants overexpressing the snapdragon *Delila (Del)* gene (Naing et al., 2017) showed enhanced anthocyanin production and improved abiotic tolerance. The results of previous studies have emphasized the importance of producing anthocyanin-enriched plants, which can provide antioxidants responsible for scavenging ROS to overcome abiotic stress conditions. Lim et al. (2016) indicated that overexpression of *RsMYB1* enhanced anthocyanin levels and antioxidant activity. Certain studies have demonstrated the role of MYB, GSH, and PCS in heavy metal tolerance in maize and walnut plants (Li et al., 2017; Xu et al., 2018); however, the yield penalty caused by the specific heavy metals was not described. Recently, Ai et al. (2017) showed that overexpression of *RsMYB1* enhanced anthocyanin accumulation in petunias. However, they did not investigate whether the anthocyanin-enriched transgenic plants expressing *RsMYB1* could tolerate heavy metal stress. Therefore, we aimed to investigate the stress tolerance of anthocyanin-enriched transgenic plants, which has not been adequately addressed to date.

In the present study, we used anthocyanin-enriched T_2_ transgenic petunia lines (PM2 and PM6) expressing *RsMYB1*, which were developed by successive pollination of the T_0_ transgenic lines reported previously (Ai et al., 2017), in order to investigate whether they are able to tolerate heavy metal stress. The tolerance of PM2 and PM6 to heavy metal stress relative to that of the wild-type (WT) plants was investigated by examining several physiological and biochemical factors. In addition, the transcript levels of genes related to metal detoxification and antioxidant activity were investigated.

## Materials and Methods

### Plant Materials

The transgenic petunia lines, PM6 and PM2, expressing RsMYB1, which were developed in our previous work (Ai et al., 2017), showed visible anthocyanin pigmentation in the whole plant; therefore, we selected these lines to be examined for heavy metal stress tolerance.

### Production of the T_2_ Generation

First, the T_0_-PM6 and T_0_-PM2 lines grown in a greenhouse were self-pollinated to obtain T_1_ lines, which were then screened visually for the anthocyanin phenotype. Second, the screened T_1_ lines were self-pollinated to obtain T_2_ seeds, and these seeds were used as the source plant material for experimentation.

### Detection of Anthocyanin Content and ROS-Scavenging Activity

To determine the anthocyanin content of the T_2_-PM6, T_2_-PM2, and WT plants, their seeds were grown in a greenhouse for 6 weeks. Six-week-old T_2_-PM6 and T_2_-PM2 plants, which showed the anthocyanin phenotype, and WT seedlings were selected for the analysis of total anthocyanin content and ROS-scavenging activity.

Total anthocyanin extraction was performed following our previously published procedure (Ai et al., 2017). Briefly, fresh leaves (fifth top leaves, ∼500 mg) were collected and ground to a fine powder, which was transferred to the extraction solution. The mixture was incubated at 4°C for 24 h and then centrifuged at 13,000 rpm and 4°C for 20 min. The anthocyanin content of the supernatant was quantified with a spectrophotometer (Shimadzu, Kyoto, Japan). For determining ROS-scavenging activity, fresh leaves (fifth top leaves, 2.0 g) were collected from T_2_-PM6, T_2_-PM2, and WT seedlings, and the activity was measured using 2,2′-azino-*bis*(3-ethylbenzothiazoline-6-sulfonic acid) diammonium salt (ABTS) and 1,1-diphenyl-2-picrylhydrazyl (DPPH) assays (Kim et al., 2014; Lim et al., 2016). Three biological samples were used for each of the T_2_-PM6, T_2_-PM2, and WT seedlings, and each measurement was repeated thrice.

### Phylogenetic Analysis of RsMYB1

The amino acid sequence of RsMYB1 was aligned with those of 35 MYB TFs involved in tolerance to different abiotic stresses (e.g., cold, salt, drought, and heavy metal stress) in several plant species using MEGA7 software; a phylogenetic tree was constructed using the maximum likelihood method. Initial trees for the heuristic search were obtained automatically by applying the neighbor-joining and BioNJ algorithms to a matrix of pairwise distances estimated using a Jones–Taylor–Thornton (JTT) model.

### *In vitro* Seed Germination and Heavy Metal Treatments

For *in vitro* seed germination, seeds of the T_2_-PM6, T_2_-PM2, and WT plants were soaked in 0.05% sodium hypochlorite (Yuhan Co., Ltd., Seoul, South Korea) containing 0.01% Tween 20 (Duchefa, Haarlem, Netherlands) for 10 min and then rinsed with sterile distilled water at least thrice. The sterilized T_2_-PM6 and T_2_-PM2 seeds were sown in Murashige and Skoog (MS) basal medium containing 3% sucrose, 1 mg⋅L^-1^ phosphinothricin (PPT), and 0.8% agar to obtain only the seedlings expressing RsMYB1; WT seeds were cultured on the same medium without PPT. The cultures were incubated at 25 ± 2°C with a 16 h photoperiod and a light intensity of 50 μmol m^-2^ s^-1^ for 30 days.

The T_2_-PM6 and T_2_-PM2 seedlings that were red and uniformly sized were selected for the heavy metal stress experiment. The T_2_-PM6, T_2_-PM2, and WT seedlings were then stressed by continuous culturing in MS liquid medium containing increasing concentrations of CuSO_4_, ZnSO_4_, K_2_Cr_2_O_7_ (25, 50, and 100 μM for each salt), or MnSO_4_ (100, 250, and 500 μM) for 10 days per concentration on a rotary shaker set to 50 rpm. The concentrations were chosen based on those used in previous studies (Baek et al., 2012; Liu et al., 2015). MS liquid medium without heavy metals was used as the control. The culture conditions were the same as described above. Each treatment contained 20 seedlings, and there were three replicates.

### Effects of Different Heavy Metals

After the plants were treated with the final concentrations of CuSO_4_, ZnSO_4_, K_2_Cr_2_O_7_ (100 μM), and MnSO_4_ (500 μM) (see section 2.5), the total time taken for the treatment periods was 30 days (from initial to final concentration treatment). At the end of the stress period, 15 plants each of T_2_-PM6, T_2_-PM2, and WT cultured in heavy metal-free media (control condition) and heavy metal-containing media (stress conditions) were randomly selected for physiological, biochemical, and molecular analyses. These analyses included plant height, fresh weight, stomatal density, fluorescence, photosynthetic pigment content, relative water content (RWC), heavy metal uptake, and gene expression. The measurements were taken thrice, and the data represent the means of three replicates.

### Measurement of RWC

Relative water content was measured using the seventh leaf from the top of T_2_-PM6, T_2_-PM2, and WT plants subjected to 30 days of heavy metal stress or control conditions. Fresh leaf weight was immediately recorded after excision from the plants. The leaves were then floated in deionized water at 4°C overnight, and their rehydrated weights were recorded. Finally, the leaves were oven-dried at 70°C overnight, and their dry leaf weight was recorded. The formula for determining RWC was as follows: RWC = (fresh weight-dry weight)/(rehydrated weight-dry weight). Five leaves each from the T_2_-PM6 and WT plants were used to determine RWC, and the analysis was repeated three times.

### Determination of Chlorophyll Content

Following the stress, chlorophyll content was measured using the fifth leaf from the top of the T_2_-PM6, T_2_-PM2, and WT plants, according to the method of Baek et al. (2012). Briefly, the leaves were homogenized in 15 mL methanol, and the homogenate was filtered through two layers of cheesecloth. This was followed by centrifugation at 3,000 *× g* for 10 min. The total chlorophyll content in the supernatant was measured and calculated using the formula described by Wellburn (1994).

### Detection of Anthocyanin Content and ROS-Scavenging Activity

Following the stress, anthocyanin content and ROS-scavenging activity (the latter using DPPH and ABTS assays) were measured using the seventh leaf from the top from the T_2_-PM6, T_2_-PM2, and WT plants. The protocols were identical to those used in the above experiment (Kim et al., 2014; Lim et al., 2016; Ai et al., 2017). Three biological samples were used for each of the T_2_-PM6, T_2_-PM2, and WT plants, and each measurement was repeated thrice.

### Determination of Stomatal Density

To determine whether the heavy metal treatments affected stomatal density in the T_2_-PM6, T_2_-PM2, and WT plants, 2-cm-long leaf segments (middle part) from the fifth leaves from the top were excised with scalpel blades. The excised leaf segments were immediately fixed in formalin–acetic acid–alcohol and kept overnight according to the protocol used by Naing et al. (2015). The samples were then dehydrated for 10 min using serial ethanol concentrations (25, 50, 70, 85, and 100%). The dehydrated samples were dried to their critical point at room temperature and then coated with gold-palladium on a Quick Cool Coater (Sanyu-Denshi, Japan). The stomatal density of each sample was examined using a scanning electron microscope (SEM; JEOL Ltd., Tokyo, Japan). Investigations were performed on three samples per treatment with three replicates.

### RNA Extraction and Gene Expression Analysis by Quantitative Reverse Transcription PCR (qRT-PCR)

The transcript levels of genes related to metal detoxification [*glutathione S-transferase (GST)* and *PCS*] and antioxidant activity [*superoxide dismutase (SOD)*, *catalase (CAT)*, and *peroxidase (POX)*] in the T_2_-PM6, T_2_-PM2, and WT plants with and without heavy metal stress were investigated. Total RNA was isolated from 100 mg of leaf tissue per treatment using the TRI Reagent^TM^ (Ambion, United States). Exactly 1 μg of total RNA and an oligo (dT)20 primer were used for reverse transcription (ReverTra Ace-α^®^, Toyobo, Japan). Then, the transcript levels of the genes (*GST*, *PCS*, *SOD*, *CAT*, *POX*, and *actin*) were measured using a StepOnePlus^TM^ Real-Time PCR system (Thermo Fisher Scientific, Waltham, MA, United States) (Naing et al., 2017). Relative gene expression was calculated using the quantitative-comparative *C*_T_ (ΔΔ*C*_T_) method. The primers and PCR conditions for the detected genes are listed in **Table 1**. Three samples per plant line were used, and the analysis was repeated three times.

**Table 1:** Primer sequences and PCR conditions used for qRT-PCR analysis in this experiment.

Genes	Accession no.	Primer sequences (5′ to 3′)	PCR conditions
*GST*	NM_001325692.1	F: CGC AAA GGA GAG GAG CAA GA R: TGT CAC CCG CAA AGA ATT TCT	95°C (10 min)-[95°C (30 s)- 60°C (30 s)] followed by 40 cycles – 95°C (15 s)- 60°C (30 s)- 95°C (15 s)


*PCS*	KP136425.1	F: GCC CAG TGT GTG GAC TTG AT R: CGA AGA GAA ATT AGG ACG TCA ACA	
*SOD*	EU342358.1	F: GCC AGC TTT GAA GAT GAA CGA R: GCC TAA TGC TCT TCC CAC CAT	
*CAT*	U93244	F: GCC AAA TCC CAA GTC CCA TA′ R: ATC GTC GAA GAG GAA AGT GAA CA	
*POX*	D11396.1	F: ACT GCT CCG TCA CCC AAA AC′ R: GCC CTG GTT GCT TAA GTC	
*Tub*	SGN-U207876	F: TGGAAACTCAACCTCCATCCA R: TTTCGTCCATTCCTTCACCTG	


### Uptake of Heavy Metals

Exactly 1 g of dried leaf tissue per treatment was collected from the T_2_-PM6, T_2_-PM2, and WT plants to determine heavy metal uptake (Cu, Zn, Mn, and Cr). The analysis was carried out as described by Cataldi et al. (2003). There were three samples per treatment and three replicates.

### Statistical Analysis

Data were collected on day 30 of the experiment and statistically analyzed using SPSS version 11.09 (IBM Corporation, Armonk, NY, United States). The results are presented as the means ± SE. Least significant difference tests were used to compare the means, and the significance was set at *P* < 0.05.

## Results

### Detection of Anthocyanin Content and ROS-Scavenging Activity

**FIGURE 1:**
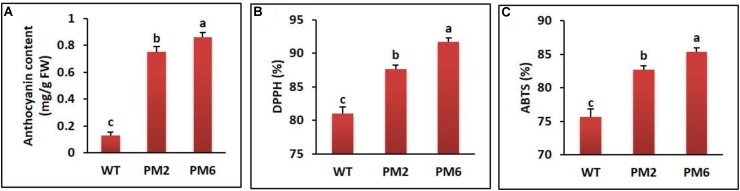
Comparison of anthocyanin content **(A)** and ROS-scavenging activities assessed by DPPH **(B)** and ABTS **(C)** assays in the T_2_-PM6, T_2_-PM2, and the wild-type (WT) plants grown in the greenhouse for 6 weeks. Error bars indicate the SEM. FW, fresh weight.

All of the homozygous T_2_-PM6 and T_2_-PM2 plants, which were obtained by successive self-pollination of T_0_ and T_1_ plants, displayed anthocyanin-containing phenotypes; the total leaf anthocyanin content in T_2_-PM6 was significantly higher than that in T_2_-PM2, followed by that in the WT plants (**Figure 1A**). To determine the association between anthocyanin content and ROS-scavenging activity, we measured the latter in the T_2_-PM6, T_2_-PM2, and WT plants using ABTS and DPPH assays. As shown in **Figures 1B,C**, the ABTS and DPPH activities were highest in the T_2_-PM6 plants, followed by T_2_-PM2 and WT plants, indicating that higher ROS-scavenging activity depends on the anthocyanin content of the plants.

### Phylogenetic Analysis of RsMYB1

**FIGURE 2:**
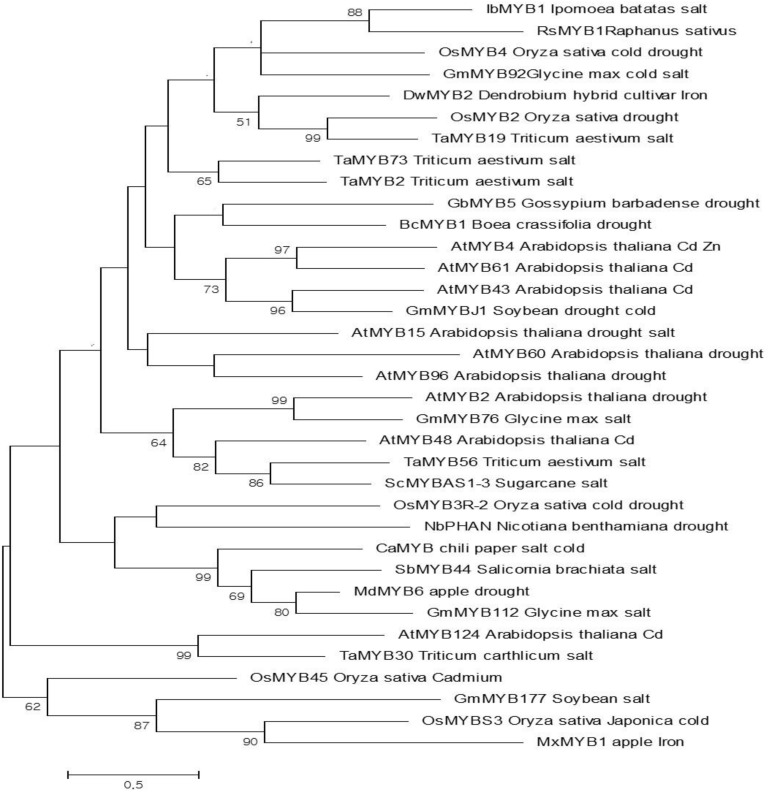
Phylogenetic relationships of the RsMYB1 transcription factor (TF) with other MYB TFs that confer tolerance to various abiotic stresses. The evolutionary history was inferred by using the maximum likelihood method based on the Poisson correction model [1]. The tree with the highest log likelihood (–4439.60) is shown. Initial tree(s) for the heuristic search were obtained automatically by applying neighbor-joining and BioNJ algorithms to a matrix of pairwise distances estimated using a JTT model and then selecting the topology with superior log likelihood value. The tree is drawn to scale, with branch lengths measured in the number of substitutions per site. The analysis involved 35 amino acid sequences. All positions containing gaps and missing data were eliminated. There were 55 positions in the final dataset. Evolutionary analyses were conducted in MEGA7 [2].

The ROS-scavenging activity experiments demonstrated that the T_2_-PM6 and T_2_-PM2 plants expressing RsMYB1 had higher-ROS scavenging activity than the WT plants. Thus, it was of interest to compare the degree of abiotic stress tolerance in the T_2_-PM6 and T_2_-PM2 plants to that in the WT plants. Before the stress treatment, we clarified the potential role of RsMYB1 in abiotic stress tolerance by building a phylogenetic tree based on the full-length amino acid sequences of 35 R2R3-MYB TFs isolated from different species that have been found to be tolerant to different abiotic stresses (cold, drought, salt, and heavy metals). The resulting tree indicated that RsMYB1 was phylogenetically related to other MYB TFs and was clustered with six TFs (IbMYB1, OsMYB4, GmMYB92, DwMYB2, OsMYB2, and TaMYB19), which confer tolerance to different abiotic stresses in various crops (**Figure 2**). This suggested that RsMYB1 has the same functional role as that of the six TFs. According to the phylogenetic tree, RsMYB1 has high sequence similarity with IbMYB1, which is associated with anthocyanin accumulation and salt tolerance.

### Assessment of Plant Growth Parameters Under Heavy Metal Stress

**FIGURE 3:**
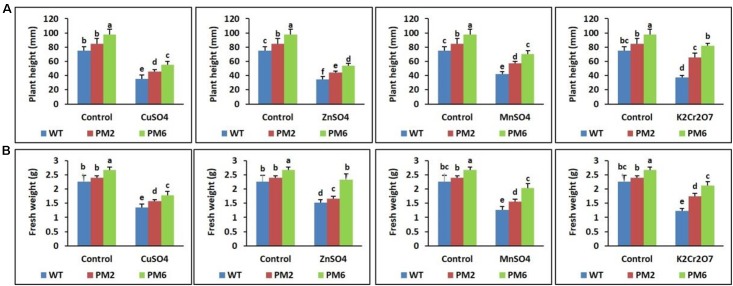
Comparisons of plant height **(A)** and fresh weight **(B)** among the T_2_-PM6, T_2_-PM2, and the WT plants treated with the indicated heavy metal salts. Data were acquired on the thirtieth day after starting the experiments. Error bars show the SEM.

The growth of the T_2_-PM6, T_2_-PM2, and WT plants in response to the stress of various heavy metals (CuSO_4_, ZnSO_4_, MnSO_4_, and K_2_Cr_2_O_7_), as well as under normal growing conditions (without heavy metals), was evaluated 30 days after the treatments began. Survival of the control plants was high, and the plants produced well-developed, broad leaves and regular roots. When the plants were treated with CuSO_4_, ZnSO_4_, K_2_Cr_2_O_7_ (25 μM each), or MnSO_4_ (100 μM) for 10 days, the growth parameters were not significantly different from those of the plants grown under normal conditions (data not shown). However, the growth parameters started to decrease when the plants were continuously treated with higher concentrations of heavy metals (50 μM of CuSO_4_, ZnSO_4_, K_2_Cr_2_O_7_, or 250 μM MnSO_4_) for another 10 days (data not shown), and they significantly decreased when the plants were subjected to the highest concentrations (100 μM of CuSO_4_, ZnSO_4_, K_2_Cr_2_O_7_, or 500 μM MnSO_4_) for an additional 10 days. The T_2_-PM6 plants were found to be more tolerant to heavy metal stress than the T_2_-PM2 plants were, followed by the WT plants (i.e., PM6 > PM2 > WT), which was similar to the growth parameter responses in the plants (i.e., PM6 > PM2 > WT) (**Figures 3A,B**). In addition, the degree of tolerance to the heavy metals varied depending on the heavy metal used. CuSO_4_ and ZnSO_4_ were found to be the most toxic to the plants, particularly the WT plants. Overall, the presence of high concentrations of different heavy metals significantly inhibited plant growth compared to that under normal growing conditions. Furthermore, more severe toxicity was clearly observed in the WT plants, followed by the T_2_-PM2 and T_2_-PM6 plants. Therefore, the anthocyanin-enriched plants had enhanced resistance to heavy metal stress. To demonstrate tolerance of the anthocyanin-enriched plants to heavy metal stress, physio-biochemical factors (e.g., chlorophyll content, RWC, anthocyanin content, ROS-scavenging activities, and stomatal density), accumulation of the heavy metals, and expression levels of genes related to metal detoxification and antioxidant activities were measured subsequently in the plants.

### Assessment of Chlorophyll Content and RWC

**FIGURE 4:**
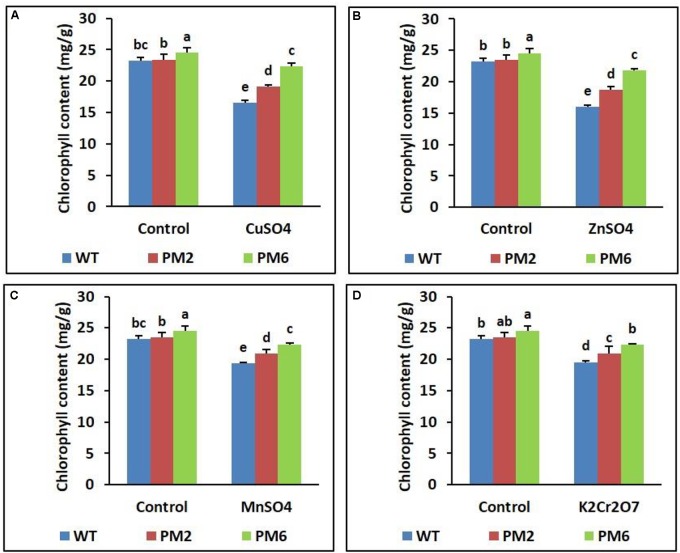
Comparison of chlorophyll content in the T_2_-PM6, T_2_-PM2, and WT plants after exposure to the indicated heavy metals (**A**, CuSO_4_; **B**, ZnSO_4_; **C**, MnSO_4_; **D**, K_2_Cr_2_O_7_). Data were acquired on the thirtieth day after starting the experiments. Error bars show the SEM.

**FIGURE 5:**
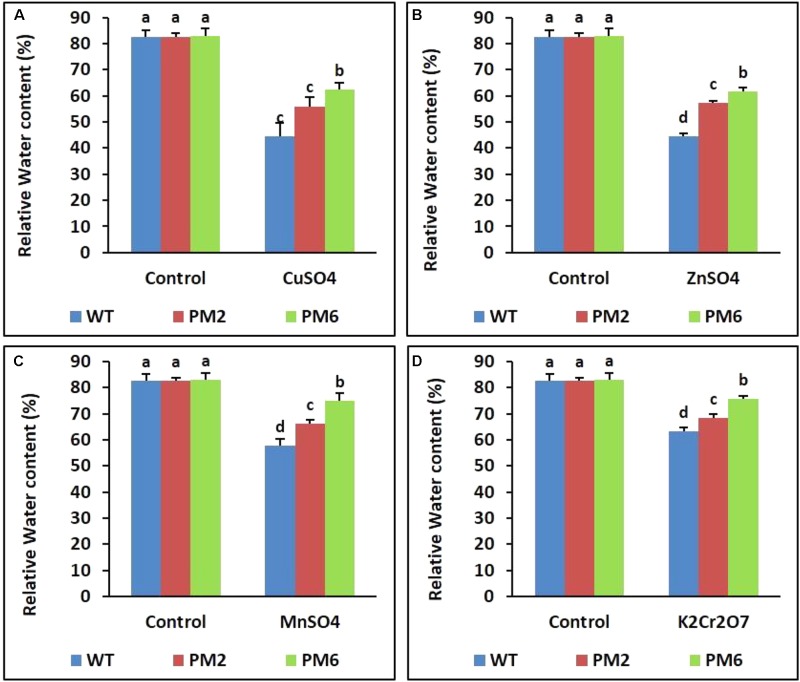
Comparison of relative water content (RWC) in the T_2_-PM6, T_2_-PM2, and WT plants after exposure to the indicated heavy metals (**A**, CuSO_4_; **B**, ZnSO_4_; **C**, MnSO_4_; **D**, K_2_Cr_2_O_7_). Data were acquired on the thirtieth day after starting the experiments. Error bars show the SEM.

Following stress, the chlorophyll content and RWC of the T_2_-PM6, T_2_-PM2, and WT plants were lower under stress conditions than under normal growing conditions (control). The levels detected in the T_2_-PM6 plants were significantly higher than those in the T_2_-PM2 followed by the WT plants, particularly under stress conditions (**Figures 4**, **5**).

### Assessment of Anthocyanin Content and ROS-Scavenging Activity

**FIGURE 6:**
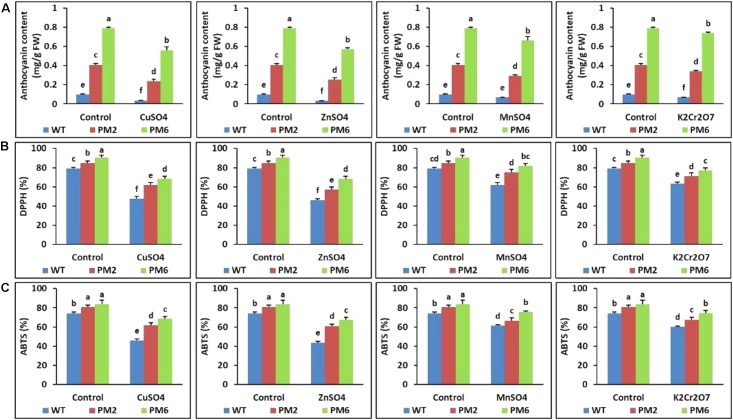
Comparison of anthocyanin content **(A)** and ROS-scavenging activities assessed by DPPH **(B)** and ABTS **(C)** assays in the T_2_-PM6, T_2_-PM2, and WT plants after exposure to the indicated heavy metals. Data were collected on the thirtieth day after starting the experiments. Error bars show the SEM.

The anthocyanin content and ROS-scavenging activity (ABTS and DPPH assays) in the T_2_-PM6, T_2_-PM2, and WT plants were measured. Significant reductions in these values were observed in all plants under stress conditions. However, a more severe reduction was observed in the WT plants than in the T_2_-PM6 and T_2_-PM2 plants (**Figures 6A–C**).

### Reduction in Stomatal Density Under Heavy Metal Stress

**FIGURE 7:**
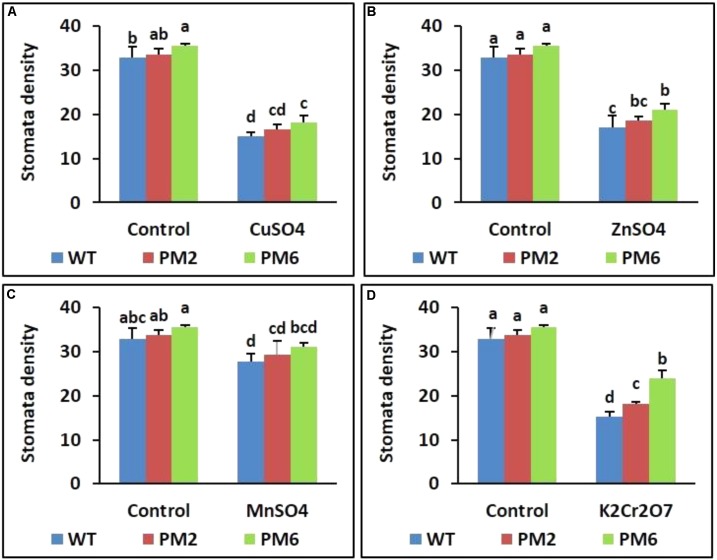
Comparison of stomatal density in the T_2_-PM6, T_2_-PM2, and WT plants after exposure to the indicated heavy metals (**A**, CuSO_4_; **B**, ZnSO_4_; **C**, MnSO_4_; **D**, K_2_Cr_2_O_7_). Data were taken on the thirtieth day after starting the experiments. Error bars show the SEM.

The stomatal densities of the T_2_-PM6, T_2_-PM2, and WT plants were investigated using an SEM to determine whether heavy metals affected stomatal density. Under normal growth conditions (control), high stomatal density was observed in the T_2_-PM6, T_2_-PM2, and WT plants. However, stomatal density decreased when the plants were exposed to the heavy metals (**Figure 7**). In addition, the extent of stomatal reduction varied depending on the type of heavy metal used.

### Expression Profiles of Genes Related to Antioxidant Activity

**FIGURE 8:**
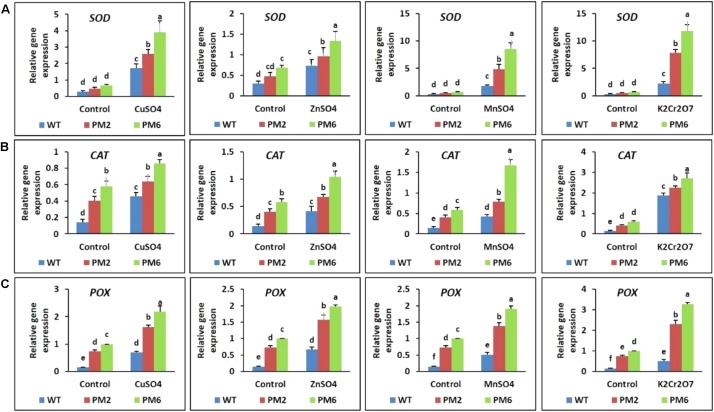
Expression analysis of the antioxidant-related genes *superoxide dismutase* [*SOD*, **(A)**], *catalase* [*CAT*, **(B)**], and *peroxidase* [*POX*, **(C)**] in the T_2_-PM6, T_2_-PM2, and WT plants after exposure to the indicated heavy metals. Data were taken on the thirtieth day after starting the experiments. Error bars show the SEM.

qRT-PCR was used to clarify the expression profile of antioxidant genes (i.e., *SOD*, *CAT*, and *POX*) in the T_2_-PM6, T_2_-PM2, and WT plants with and without heavy metal treatment. The results show that the stress treatments increased the transcript levels of the tested genes in all plants compared with those under normal growing conditions (control). However, the genes were more highly expressed in T_2_-PM6, followed by T_2_-PM2, and then WT plants under stress conditions with all heavy metals (**Figures 8A–C**). Therefore, the expression levels of the genes paralleled the degree of tolerance of the plants to heavy metal stress.

### Expression Profiles of Genes Related to Metal Detoxification

**FIGURE 9:**
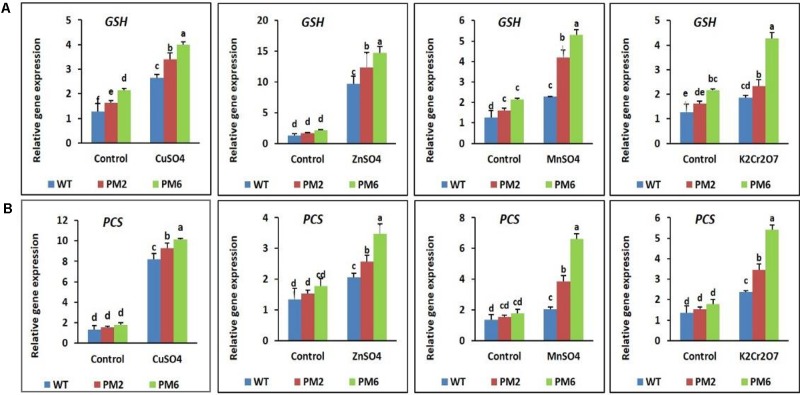
Expression analysis of the heavy metal stress tolerance genes *glutathione S-transferase* [*GST*, **(A)**] and *phytochelatin synthase* [*PCS*, **(B)**] in the T_2_-PM6, T_2_-PM2, and WT plants after exposure to the indicated heavy metals. Data were taken on the thirtieth day after starting the experiments. Error bars show the SEM.

The expression levels of genes related to metal detoxification (i.e., *GST* and *PCS*), which are normally regulated by heavy metal stress, were analyzed by qRT-PCR. When the plants were subjected to heavy metal stress, their expression patterns of *GST* and *PCS* were similar to the antioxidant gene patterns described in section 3.7. Expression of the genes in the T_2_-PM6, T_2_-PM2, and WT plants was low under normal growth conditions, but their expression increased when the plants were exposed to heavy metal stress. However, the stress-induced increases in expression were higher in the T_2_-PM6 plants, followed by the T_2_-PM2 and WT plants (**Figures 9A,B**), which was consistent with the degree of heavy metal tolerance of the plants (i.e., T_2_-PM6 > T2-PM2 > WT).

### Accumulation of Heavy Metals in the T_2_-PM6, T_2_-PM2, and WT Plants

**FIGURE 10:**
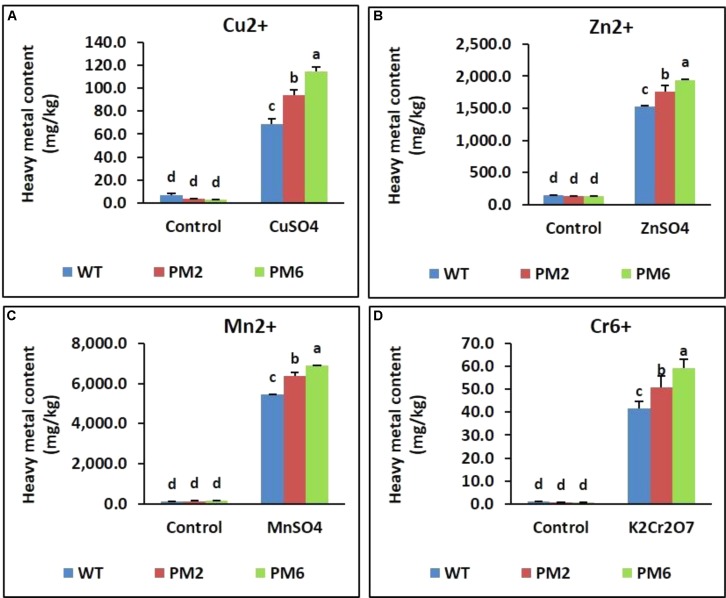
Heavy metal content [Cu^2+^ **(A)**, Zn^2+^ **(B)**, Mn^2+^ **(C)**, and Cr^6+^ **(D)**] accumulated in leaf tissue following heavy metal-stress treatment of the T_2_-PM6, T_2_-PM2, and WT plants. Data were taken on the thirtieth day after starting the experiments. Error bars indicate the SEM.

Under normal growth conditions, the heavy metal content of the T_2_-PM6, T_2_-PM2, and WT plants was quite low, and the three plant types did not significantly differ in their content. However, when the plants were exposed to different heavy metals, metal uptake significantly increased, but the total content of detectable metals per plant was significantly higher in the order T_2_-PM6 > T_2_-PM2 > WT (**Figure 10**).

Taken together, overexpression of *RsMYB1* in transgenic petunias enhanced anthocyanin content, which led to higher antioxidant activity to scavenge the ROS induced by heavy metal stress. Moreover, higher expression levels of genes related to metal detoxification and antioxidant activity were detected in the transgenic lines expressing *RsMYB1*. The sequence of *RsMYB1* is similar to that of other MYBs that confer tolerance to various abiotic stresses. We suggest that the transgenic lines have advantages that impart a greater capability to tolerate heavy metal stress when the plants are treated for 30 days.

## Discussion

Anthropogenic activities have led to a continuous increase in heavy metal contamination of agricultural soil. This is becoming a major concern worldwide (Rascio and Navari-Izzo, 2011; Villiers et al., 2011) because these contaminated soils negatively affect plant physiological processes through the generation of ROS, which results in lower crop yields (DalCorso et al., 2008; Hossain et al., 2009, 2010; Rascio and Navari-Izzo, 2011; Villiers et al., 2011). Therefore, heavy metal toxicity and the defense mechanisms used by plants to scavenge ROS and detoxify heavy metals need to be elucidated (Rascio and Navari-Izzo, 2011). Previous studies have shown that antioxidants are involved in the scavenging of ROS generated by heavy metal stress (Hirschi et al., 2000; Mittler et al., 2004; Tseng et al., 2007; Ai et al., 2018). In addition, the roles of GSH and *PCS* in the detoxification of heavy metals and ROS scavenging have been documented (Millar et al., 2003; Freeman et al., 2004; Foyer and Noctor, 2005; Hirata et al., 2005; Shao et al., 2008). The enhancement of antioxidant activity and stress tolerance in anthocyanin-enriched plants has been reported for various plant species (Winkel-Shirley, 2002; Dixon et al., 2005; Agati et al., 2011; Dehghan et al., 2014; Ai et al., 2018). Recently, the overexpression of MYB TFs in various species has been shown to enhance anthocyanin accumulation and abiotic stress tolerance (Cheng et al., 2013; Meng et al., 2014; Qi et al., 2015; Yuan et al., 2015). In our previous study, the petunia transgenic lines, PM6 and PM2, which express RsMYB1, distinctly and consistently enhanced anthocyanin accumulation (Ai et al., 2017); however, we did not investigate whether these lines were tolerant to heavy metal stress.

Before commencing the stress experiment, we produced homozygous T_2_-PM6 and T_2_-PM2 plants by successive self-pollination of T_0_ and T_1_ plants. The T_2_-PM6 and T_2_-PM2 plants showed stable anthocyanin-containing phenotypes and contained higher anthocyanin levels and ROS-scavenging activities (as assessed by ABTS and DPPH assays) than did the WT plants, suggesting that the higher anthocyanin accumulation is linked to greater ROS-scavenging activity, as has been reported previously (Fini et al., 2011; Nakabayashi et al., 2014). Moreover, these results support the findings of Lim et al. (2016), who reported that RsMYB1 overexpression in *Arabidopsis* strongly enhances anthocyanin production and promotes ROS-scavenging activity; however, the authors did not examine the role of transgenic *Arabidopsis* plants overexpressing RsMYB1 in protecting against abiotic stress conditions. According to our phylogeny results, the sequence of RsMYB1 is phylogenetically related to six MYBs (i.e., IbMYB1, OsMYB4, GmMYB92, DwMYB2, OsMYB2, and TaMYB19), which confer tolerance to various abiotic stresses in these crops. Owing to the presence of high anthocyanin levels and ROS-scavenging activity, as well as sequence similarity with other MYBs that confer tolerance to abiotic stress, we wanted to investigate the heavy metal stress tolerance of the T_2_-PM6, T_2_-PM2, and WT plants by measuring physiological and biochemical parameters and expression levels of genes involved in the tolerance to heavy metal stress.

In this study, the T_2_-PM6, T_2_-PM2, and WT plants survived under normal growth conditions and their growth parameters (i.e., plant height, root length, and fresh weight) were not significantly different. However, when they were exposed to different heavy metal salts (i.e., CuSO_4_, ZnSO_4_, MnSO_4_, or K_2_Cr_2_O_7_), signs of plant growth inhibition, water deficiency, and chlorophyll degradation were observed, which were more severe in the WT plants. This was corroborated with the data showing reduced chlorophyll and RWC in the T_2_-PM6, T_2_-PM2, and WT plants (with respect to reductions, WT > T_2_-PM2 > T_2_-PM6). It seems that heavy metal stress reduced the formation of roots, which take up water, and interrupted the synthesis of chlorophyll, which is required for plant photosynthesis, leading to the reduction in RWC and chlorophyll content. Among the heavy metals tested, the toxicity caused by CuSO_4_ and ZnSO_4_ was more severe than that of the others, because leaf chlorosis and plant growth inhibition were more severe in the plants treated with these metals. The effects of CuSO_4_ and ZnSO_4_ exposure in this study confirmed the results previously reported by Ebbs and Kochian (1997) and Lewis et al. (2001). Heavy metals have been reported to be toxic to plants and cause injuries through the generation of ROS, which disturb physiological functions (Ebbs and Kochian, 1997; Lewis et al., 2001; Sharma et al., 2003; Scoccianti et al., 2006). However, in this study, the degree of tolerance to heavy metals was T_2_-PM6 > T_2_-PM2 > WT plants, which is probably due to the greater ROS-scavenging ability (determined via DPPH and ABTS assays) in the anthocyanin-enriched plants (T_2_-PM6 > T_2_-PM2) than in the WT plants. The role of anthocyanin-induced ROS-scavenging activity in the tolerance to different abiotic stresses has been established in previous studies (Hossain et al., 2009; Cheng et al., 2013; Nakabayashi et al., 2014; Qi et al., 2015; Yuan et al., 2015). In addition, the presence of higher RWC in the T_2_-PM6 and T_2_-PM2 plants than that in the WT plants could be caused by higher ROS-scavenging activities in the former plants, because these activities can detoxify heavy metals accumulated in the roots, allowing the roots to take up water easily (Hirschi et al., 2000; Tseng et al., 2007). However, ROS-scavenging activity and anthocyanin content were also lower in the plants under stress conditions than in the plants under normal growing conditions. This outcome might be due to antioxidant activity to defend against metal-induced ROS stress. In addition, the occurrence of higher toxicity of heavy metals in the WT than in the T_2_-PM6 and T_2_-PM2 plants could be caused by the absence of sufficient anthocyanin, which scavenges ROS, suggesting that high ROS-scavenging activity is necessary to counter heavy metal-induced stress.

Theoretically, stomata play a critical role in photosynthesis because they are responsible for uptake of carbon dioxide from the atmosphere and release of oxygen. In this study, inhibition of plant growth under heavy metal stress was associated with a reduction in stomatal density. This suggests that the stomatal density reduction caused by heavy metal stress would decrease leaf photosynthesis, thereby reducing plant growth. These results support the findings reported by Yilmaz et al. (2009) and Kambhampati et al. (2005), who also reported that heavy metals reduced stomatal density.

The expression levels of the antioxidant genes (*SOD*, *CAT*, and *POX*) responsible for scavenging or neutralizing ROS were investigated to reveal the mechanisms underlying the higher tolerance of the plants to heavy metal stress (T_2_-PM6 > T_2_-PM2 > WT plants). The expression levels of these genes were significantly higher under heavy metal stress than under normal growth conditions for the T_2_-PM6, T_2_-PM2, and WT plants. This might occur because the plants enhanced gene expression to defend against ROS formation caused by the heavy metals. This supports the findings of previous studies (Singh et al., 2013; Bashri and Prasad, 2015). However, the greater inhibition of WT growth compared with that of the T_2_ plants (T_2_-PM6 > T_2_-PM2) suggests that the induction of antioxidant activity was significantly lower in the WT plants than in the T_2_ plants. Furthermore, gene upregulation in the WT plants seems to have been insufficient to properly scavenge ROS. Under the same conditions, the higher gene expression in the T_2_ plants than that in the WT plants could be explained by the presence of anthocyanin, which is regulated by RsMYB1, or by RsMYB1 directly binding to the proteins involved in antioxidant production. High anthocyanin accumulation is linked to high antioxidant activity (Winkel-Shirley, 2002; Dixon et al., 2005; Agati et al., 2011; Dehghan et al., 2014; Naing et al., 2017). Therefore, these results support the hypothesis that the degree of stress tolerance in the plants (T_2_-PM6 > T_2_-PM2 > WT) depends on their antioxidant content.

The roles of other genes, such as *GST* and *PCS*, which are involved in antioxidant defense mechanisms and heavy metal detoxification, were also investigated. *GST* and *PCS* expression levels increased in response to heavy metal stress in the T_2_-PM6, T_2_-PM2, and WT plants. Furthermore, their expression levels were higher in the T_2_ plants (T_2_-PM6 > T_2_-PM2) than in the WT plants; this corresponds with the degree of tolerance to heavy metals in the former plants (i.e., T_2_-PM6 > T_2_-PM2 > WT). Thus, it is likely that RsMYB1 directly binds to the proteins involved in metal detoxification. The high expression levels of both genes suggest that they play major roles in scavenging ROS, detoxifying xenobiotics and heavy metals, and maintaining ionic homeostasis (Freeman et al., 2004; Foyer and Noctor, 2005; Hirata et al., 2005; Shao et al., 2008). Overexpression of these genes in other plants (e.g., *Brassica juncea*, *Arabidopsis*, *Populus canescens*, and *Nicotiana tabacum*) has also been found to enhance tolerance to heavy metal stress (Bittsánszkya et al., 2005; Cairns et al., 2006; Singla-Pareek et al., 2006; Gasic and Korban, 2007a,b). In this study, despite enhanced expression of the *GST* and *PCS* genes under heavy metal stress, there was a reduction in plant growth when the plants were exposed to heavy metals, which indicates that the *GST* and *PCS* expression levels were insufficient to defend completely against heavy metal stress, particularly in the WT plants. This strongly suggests that the heavy metal tolerance mechanism requires high GST and PCS enzymatic activity.

More heavy metals accumulated in the T_2_ plant shoots than in the WT plant shoots. This also suggests that the T_2_ plants were more tolerant to heavy metals than the WT plants, which led to the increased uptake of heavy metals. Another explanation for this observation is that the roots of the T_2_ plants were less damaged by the metals than the WT roots were because of increased anthocyanin levels (higher ROS-scavenging activity) and higher expression levels of antioxidant (*SOD*, *CAT*, and *POX*) and metal detoxification genes (*GST* and *PCS*). This would have led to greater alleviation of the negative effects of metal stress on root water uptake, because the RWC detected in the T_2_ plants was higher than that in the WT plants. These results support the findings of Zhu et al. (1999) and Wawrzyński et al. (2006), who reported that plants expressing *PCS* and GSH synthetic genes showed enhanced Cd accumulation and tolerance. Bennett et al. (2003) also claimed that overproduction of GSH in mustard led to accumulation of 2.4- to 3-fold more Cr, Cu, and Pb than that in WT plants.

## Conclusion

RsMYB1 has been found to play a regulatory role in anthocyanin accumulation in petunias. According to phylogenetic analysis, it has sequence similarity with other MYBs that confer tolerance to various abiotic stresses. However, its regulatory role in the expression of genes related to antioxidant activity and metal detoxification during abiotic stress was not known. Therefore, in this study, we characterized the functional involvement of RsMYB1 in tolerance to heavy metal stress using RsMYB1-overexpressing plants (T_2_-PM6 and T_2_-PM2 plants) and WT plants. The results suggest that T_2_-PM6 and T_2_-PM2 plants are more capable of tolerating heavy metal stress because RsMYB1 induces higher expression levels of genes related to metal detoxification and antioxidant activity. Therefore, *RsMYB1* could be exploited as a dual function gene that will improve anthocyanin production and heavy metal stress tolerance in horticultural and agricultural crops.

## Author Contributions

AN and TA designed the study, conducted the experiments, and wrote the manuscript. SL assisted in the conduction of the experiments. CK and B-WY supervised the experiments at all stages and performed the critical revisions of the manuscript. All authors read and approved the final manuscript.

## Conflict of Interest Statement

The authors declare that the research was conducted in the absence of any commercial or financial relationships that could be construed as a potential conflict of interest.
